# Measuring and Modifying Threat-Related Attention Bias in Posttraumatic Stress Disorder: An Attention Bias Modification Study

**DOI:** 10.1155/2024/3683656

**Published:** 2024-10-04

**Authors:** Mikael Rubin, Andrea Niles, Travis Evans, Paige Tripp, Thomas C. Neylan, Joshua Woolley, Aoife O'Donovan

**Affiliations:** ^1^Department of Psychology, Palo Alto University, Palo Alto, California, USA; ^2^Department of Psychiatry and Behavioral Sciences, University of California San Francisco, San Francisco, California, USA; ^3^San Francisco Veterans Affairs Health Care System, San Francisco, California, USA; ^4^VA Boston Healthcare System, Boston, USA; ^5^Department of Psychiatry, Boston University School of Medicine, Boston, USA

**Keywords:** attention bias, attention bias modification, PTSD, response-based computation

## Abstract

**Background:** Dominant models of posttraumatic stress disorder (PTSD) implicate threat-related attention biases in both the development and maintenance of posttraumatic stress symptoms. However, the ability to better understand and modify threat-related attention biases in PTSD has been hampered by the low reliability of attention bias measures more generally.

**Methods:** The current study adopts a new approach to calculate attention bias from a dot-probe task, response-based attention bias (RB-AB) computation, in a sample of 689 individuals reporting significantly elevated PTSD symptoms who participated in a clinical trial of threat-related attention bias modification training.

**Results:** RB-AB is a reliable strategy for deriving threat-related attention bias scores that correlate with both PTSD severity and anxiety. On the other hand, scores from the traditional approach were unreliable and not associated with clinical symptoms. Attention training led to reductions in RB-AB indices of attention bias, but not the traditional index, although attention bias training conditions did not appear to moderate these effects.

**Conclusions:** Taken together, these findings support evidence that threat-related attention biases may be a feature of PTSD and that RB-AB computation is a more reliable and valid approach for studying reaction-time-based attentional processes. Using the RB-AB approach to assess attention bias could allow us to better understand threat-related attention biases in PTSD and to ultimately develop more precise interventions to reduce threat-related attentional biases in PTSD and other disorders.

## 1. Introduction

Posttraumatic stress disorder (PTSD) is a prevalent mental health concern characterized by a broad range of symptoms that can emerge following a traumatic event [1], including four core domains: intrusions (involving intrusive thoughts and memories of the traumatic event); avoidance (actively avoiding reminders of the trauma); negative alterations in cognitions and mood (persistent negative thoughts and feelings stemming from the traumatic event); alterations in arousal and reactivity (marked changes in arousal and reactivity associated with the trauma, like hypervigilance or exaggerated startle response); and dissociation (experiencing detachment or estrangement from oneself or surroundings), which is not a required symptom cluster of DSM-5. Threat-related hypervigilance (part of alterations in arousal and reactivity) and avoidance are core features of PTSD [2].

There is evidence linking PTSD with threat-related attention biases [3], particularly with greater vigilance toward perceived threatening stimuli [4], but also with greater avoidance of perceived threatening stimuli [5]. Expanding on prior work, some research has investigated moderators of attention bias in PTSD, finding that attentional control may play an important role; specifically, greater PTSD symptoms and lower attentional control were associated with greater attentional avoidance [6]. Other work has examined attention bias and PTSD with comorbid concerns, including chronic pain [7] and borderline personality disorder [8]. In addition to cross-sectional evidence linking attention bias to PTSD, there has also been work suggesting that attentional avoidance may be a risk factor for developing PTSD [9]. Accumulating evidence implicating attentional biases in the development and maintenance of PTSD raised the potential for targeting this mechanism in treatment.

During the past decade, there has been a steady trickle of studies that purport to modify attention biases and improve symptoms of psychiatric disorders through computer-based attention bias modification (ABM) programs [10, 11]. These studies offered the exciting possibility that low-cost and highly scalable interventions could reduce psychiatric symptoms, including PTSD symptoms. However, while small studies offered promising initial findings of reduced PTSD symptoms following ABM [12], control conditions have often had similarly beneficial results as active treatment conditions or even outperformed them [13–15]. Moreover, attempts to optimize ABM programs and to understand mechanisms underlying ABM have been hampered by an inability to detect changes in the target mechanism of threat-related attention bias. As a result, there are growing concerns about the validity of attention bias measures in general and ABM interventions in particular.

Although individual studies have reported threat-related attention biases in PTSD compared to control groups, a recent large-scale meta-analysis on ABM studies did not find evidence of an attentional bias within individuals with clinical anxiety and PTSD at baseline [16]. All participants (*n* = 1005) were enrolled in ABM studies for a range of clinical concerns, and the mean bias index within/across individuals was not different from zero (i.e., on average individuals did not demonstrate an attentional bias). Thus, the meta-analysis highlighted a critical concern regarding the lack of internal consistency (measured using Cronbach's alpha and split-half reliability) of traditional metrics used to evaluate attention bias [17, 18]. Traditionally, attention bias has been measured using a dot-probe task in which participants respond to a cue that replaces either a perceived threatening or neutral stimulus across a series of trials. Scores are typically calculated as the difference between mean reaction time (RT) to response cues replacing perceived threatening stimuli and mean RT to response cues replacing neutral stimuli. Although reliability is not reported in all studies, traditional metrics of attention bias indices typically exhibit unacceptably low reliability [19]. Importantly, however, studies using alternative methods, such as event-related electroencephalography clearly demonstrate robust patterns of attentional bias despite RT measures having poor reliability [20]. Therefore, inconsistent findings of attentional bias in PTSD may not reflect an absence of attentional bias per se but instead the poor psychometric properties of traditional RT metrics used to detect attentional biases [19].

Given that RT measures provide a low-cost and feasible method to measure attentional bias, there has been a movement to develop alternative methods for calculating RT-based measures of attention bias that exhibit sufficient reliability for clinical research. Broadly, these alternative methods focus on quantifying the dynamic variability in attention biases across individual trials rather than averaging across trials as in traditional approaches [21, 22]. In particular, these methods attempt to capture the tendency of individuals with PTSD to exhibit variability in attentional bias that might include both vigilance toward threat on some trials as well as avoidance away from threat on other trials, which is obfuscated by traditional approaches that average across trials [23]. Moreover, a meta-analysis has confirmed the presence of a small association between attention bias variability and symptoms (*r* = 0.21; [24]). While reliability indices for these new measures have been better, they remain variable and do not reach traditionally acceptable thresholds, and there are additional concerns about whether these computational approaches actually measure attention bias [25]. Thus, there remains a significant need to test more consistently reliable approaches for assessing attention bias.

A recently developed response-based (RB) computational approach has yielded RT-based measures of attention bias that exhibit high reliability and appear theoretically, methodologically, and empirically sound [26]. Unlike traditional computational approaches that produce a single measure of *overall* vigilance or avoidance across trials, RB computation captures the degree to which individuals exhibit vigilance or avoidance across individual trials to produce *separate* measures of vigilance and avoidance. To this end, RB computation compares trial-level RTs to each individual's reference condition (e.g., mean neutral RT) to capture the degree to which individuals exhibit vigilance or avoidance on a given trial. By separately measuring vigilance and avoidance across trials, RB computation offers a method to disentangle dynamic fluctuations in attention bias and to quantify the relative magnitude in attentional bias patterns (i.e., vigilance relative to avoidance). Compared to traditional metrics, RB measures exhibit robust reliability and detect unique associations with anxiety symptoms [26]. Moreover, compared to traditional metrics, RB measures delineate unique neural mechanisms underlying distinct patterns of threat-related attention bias in other clinical populations [27]. Therefore, RB computation may offer a potential opportunity to disambiguate mixed findings of attention bias in PTSD. Nonetheless, to our knowledge, the RB attention bias (RB-AB) approach has not been deployed to measure attention bias in PTSD or to test the effects of ABM on attentional processes.

In the current study, we conducted secondary analyses that utilized RB-AB data from a large clinical trial, testing the efficacy of ABM for PTSD. The overarching aim of this current study was to test whether the RB-AB approach to scoring the dot-probe task yields scores with improved reliability and clinical validity compared to traditional approaches. We hypothesized that (1) RB-AB measures would exhibit greater split-half reliability than traditional AB across baseline, posttreatment and follow-up sessions; (2) greater RB-AB would be meaningfully associated with greater PTSD symptom severity and trait anxiety at the baseline session; and (3) that ABM would lead to greater reductions in RB-AB for the active ABM conditions compared to the placebo condition.

## 2. Methods

### 2.1. Participants

Participants were 689 adults (aged 18–69 years, 79.5% female, 57.8% White, and 50.4% had completed 4-year college or graduate school) who enrolled in a clinical trial of ABM that was advertised widely via the internet, with word stimuli for the treatment of PTSD and completed the baseline assessment. Participants in the trial demonstrated clinically significant PTSD symptoms (PTSD checklist scores ≥ 33, which is the recommendation by the National Center for PTSD (2018) and were randomized to a placebo, nonpersonalized, or personalized word condition. In the placebo condition, both words were neutral words (e.g., “Theater,” “Ballot,” and “Tourist”); in the nonpersonalized condition, 60 threat words (e.g., “Terror,” “Shot,” and “Toxic”) were randomly selected from a pool of 453 words, and in the personalized word condition an algorithm selected 60 words from the same pool of 453 words that were predicted to be perceived as highly threatening based on participant ratings of 55 words. In the active intervention conditions, the threat word replaced the cue 100% of the time. Participants completed the self-report assessments (baseline, posttreatment, and follow-up), attention assessments (baseline, mid-treatment, posttreatment), and training (12 sessions) remotely on their smartphones. A detailed description of the clinical trial and ABM procedures is described in the original manuscript [28].

### 2.2. Measures

#### 2.2.1. PTSD Checklist DSM-5 (PCL-5)

The PCL-5 [29] is a 20-item self-report measure for evaluating symptoms of PTSD. It has been well-validated for measuring PTSD symptoms [29]. The measure asks participants to report PTSD symptom severity on a scale from 0 (not at all) to 4 (extremely). Summed scores were used for evaluating overall PTSD symptom severity at baseline. The internal consistency for the PCL-5 was 0.92 at baseline, 0.96 postintervention, and 0.95 at follow-up,

#### 2.2.2. State-Trait Anxiety Inventory-Trait Version (STAI-T)

The STAI-T [30] is a 20-item self-report measure for assessing symptoms of anxiety. The measure asks participants to report anxiety symptoms on a scale from 1 (almost never) to 4 (almost always). The STAI-T has been frequently used as an outcome for ABM [31] and was included in the original analyses. The internal consistency for the STAI-T was 0.90 at baseline, 0.90 postintervention, and 0.91 at follow-up,

#### 2.2.3. Attention Bias

**Figure 1:**
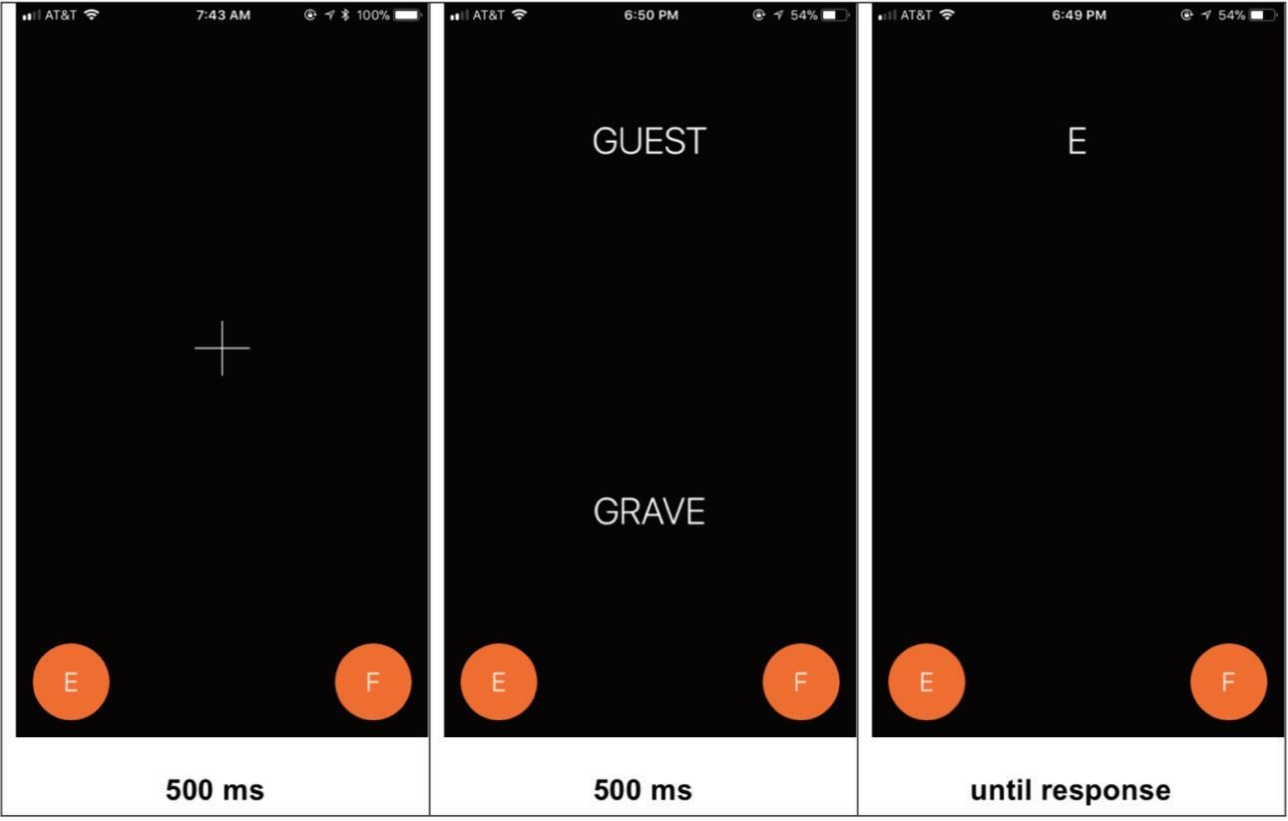
The image above shows one “trial” of the attention bias modification paradigm as it was deployed. The participant first saw a fixation cross for 500 ms. Next, two-word stimuli (threat and neutral or neutral and neutral, depending on the condition) were presented for 500 ms. Finally, either the letter “E or “F” replaced the one-word stimulus and remained on the screen until the participant identified the letter by pressing on the “E” or “F” icons on the screen of their device. The application was made available and deployed only on Apple iPhones.

The dot-probe paradigm was used to assess attention bias. The dot-probe was administered using a mobile app called “Resolving Psychological Stress” developed for the clinical trial, see Figure 1. The dot-probe assessment had participants view a fixation cross for 500 ms. After the fixation cross disappeared when the time elapsed, two words appeared on the top and bottom of the screen (one threat and one neutral). Threat words were previously rated as highly threatening in a sample of 1112 trauma-exposed individuals [32]. On the screen, the words were positioned 3 cm apart. The two words were visible for 500 ms. After this, one of the words was replaced by an “E” or “F” probe. Participants were instructed to respond to the letter that appeared using a touch-response on the bottom of the screen. Participants needed to make a response in order to continue to the next trial. The probe (letter) replaced the neutral word 50% of the time and the threat word 50% of the time. Trials, where the probe replaced the neutral word, are considered congruent, and trials where the probe replaced the threat word are considered incongruent. There were a total of 70 trials; trial order/word was randomized and counterbalanced for probe letter and location.

### 2.3. Data Preparation

#### 2.3.1. Raw RT Data Cleaning

Raw RT data were cleaned using the same procedure as previous research using RB computation, with incorrect responses, RTs shorter than 150 ms or longer than 2000 ms, as well as RTs greater or less than 2.5 standard deviations of a participant mean for each trial type being excluded [26]. Participants with fewer than 70% of trials remaining after data cleaning were excluded from further analyses (*n* = 30). See Table 1 for a demographic summary of the remaining participants.

#### 2.3.2. RB Computational Approach

In the current study, we used RB computation of attention bias based on the approach originally outlined by Evans and Britton [26]. Broadly, for each individual, we separately indexed trial-level RTs on congruent trials against the mean of incongruent trials (e.g., Incongruent_Mean −_ Congruent_Trial1_). Using this approach, positive trial-level difference scores indicate vigilance toward the threat, whereas negative trial-level difference scores indicate avoidance away from the threat. Finally, difference scores for vigilant-coded and avoidance-coded trials are separately averaged. The absolute value of the avoidance values was taken to facilitate using the lognormal family of distributions most appropriate to these data (as detailed below).

#### 2.3.3. Traditional Attention Bias Approach

Following raw RT data cleaning, traditional attention bias was computed among the 659 participants who were also included in the RB-AB analyses. Traditional AB was calculated by taking the mean difference of the incongruent trials from congruent trials for each individual.

### 2.4. Data Analysis

#### 2.4.1. Reliability

Cronbach's alpha was calculated for a range of bin numbers for the 70 trials (2–20; 2, 4, 8, 12, 16, 20), where each bin number reflected an increasingly larger number of bins used to calculate the attention bias indices (as number of bins increase, number of trials decrease). Split-half reliability was calculated by splitting the total number of trials in half (odd trials vs. even trials) for each participant. Functions from the *psych* package were used to calculate Cronbach's alpha and split-half reliability. STATA was used to calculate the internal consistency of the self-report measures.

#### 2.4.2. Modeling Approach

All analyses were conducted using a Bayesian approach implemented in the R statistical software environment using the *brms* package [33]. To evaluate the association between RB-AB, PTSD symptoms, and trait anxiety, a series of regression models were conducted with each RB-AB index (vigilance, avoidance) as a separate dependent variable and PTSD symptoms or trait anxiety as predictors. Additionally, each of the four PTSD clusters (B, C, D, and E) were entered into an exploratory multiple regression analysis predicting each RB-AB index separately. To test ABM-related changes in RB-AB, a multilevel model was conducted with the time (baseline, mid-treatment, posttreatment) by condition (placebo, nonpersonalized, personalized) interaction as the predictors of each RB-AB index and a random intercept for each individual. The placebo condition and baseline assessment served as the reference variables in all analyses where they were included. For RB-AB indices as the dependent variable, a lognormal family distribution was used to address the fit of the data to the posterior distribution based on posterior predictive checks. Priors were centered on zero with reasonable standard deviation estimates. Continuous predictors were scaled across all analyses. The models all converged (trace plots and effective sample sizes were appropriate), and posterior predictive checks indicated that the models fit the data well. The R syntax used for Bayesian analyses is available at this link: https://osf.io/kv23r/?view_only=e9b13c9871cb4131b106c8c7f7bfccba. Data are available on request from the senior corresponding author.

## 3. Results

Summaries of the mean RT indices across conditions and assessment are in Table 2; there were no differences among the groups for sociodemographic variables described in Table 1 (*p*'s > 0.05).

### 3.1. Traditional AB Approach

#### 3.1.1. Reliability of Traditional AB

Consistent with previous research, the split-half reliability of the traditional AB index was low and unacceptable at all assessments: 0.19, 0.15, 0.19. Similarly, the range for Cronbach's alpha values across bins was unacceptable for all assessments for traditional AB (baseline *α* = 0.18–0.35; mid-treatment *α* = 0.15–0.31; and posttreatment *α* = 0.19–0.38).

#### 3.1.2. Validity of Traditional AB—Associations With Baseline Symptom Severity

**Figure 2:**
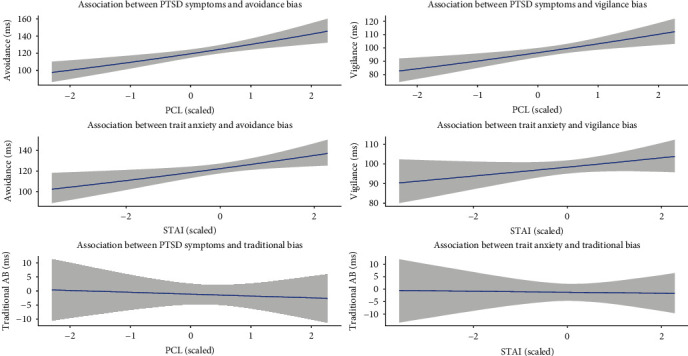
Associations between PTSD symptoms and trait anxiety with vigilance, avoidance, and traditional bias measures. *Note*: All measures on the *X*-axis are standardized (*z*-scores); STAI, state-trait anxiety inventory; PCL = PTSD checklist.

As shown in Figure 2, there was no association between traditional attention bias and PTSD symptoms, *b =* −0.66, 95% confidence Interval (CI) [−4.64, 3.30], Bayes *R*^2^ = 0.002 or trait anxiety *b* = −0.18, 95% CI [−3.50, 3.18], Bayes *R*^2^ = 0.002. There was also no main effect of time for traditional AB scores at mid-treatment *b* = −1.47, 95% CI [−5.79, 2.92] or posttreatment *b* = −1.77, 95% CI [−6.34, 2.84].

#### 3.1.3. Change in Traditional AB by Time and Treatment Condition

There was no interaction between the effect of the personalized intervention condition and time at mid-treatment, *b* = −1.95, 95% CI [−12.34, 8.55] or posttreatment *b* = −7.19, 95% CI [−18.04, 3.82], or of the nonpersonalized intervention condition on traditional AB at mid-treatment, *b* = 2.92, 95% CI [−7.75, 13.72] or posttreatment, *b* = 9.58, 95% CI [−1.78, 20.92].

### 3.2. RB-AB Approach

#### 3.2.1. Reliability of RB-AB

Split-half reliability of RB-AB was approaching acceptable or acceptable across assessments for avoidance (0.68, 0.61, 0.72) and for vigilance (0.67, 0.69, 0.71). Across bin sizes, Cronbach's alpha ranges for avoidance were (baseline *α* = 0.62–0.81; mid-treatment *α* = 0.60–0.82; and posttreatment *α* = 0.72–0.83) and ranges for vigilance were (baseline *α* = 0.66–0.82; mid-treatment *α* = 0.68–0.86; and posttreatment *α* = 0.71–0.84).

#### 3.2.2. Validity of RB-AB—Associations With Baseline Symptom Severity

Figure 2 depicts the associations between RB-AB measures and baseline symptoms. Higher levels of PTSD severity were associated with greater RB-AB avoidance scores, *b* = 0.09, 95% CI [0.04, 0.13], Bayes *R*^2^ = 0.02, indicating that for each standard deviation increase in PTSD symptom severity, there was a 9% or ~10 ms increase in the magnitude of RB-AB avoidance scores. Higher PTSD severity was also associated with greater vigilance scores, *b* = 0.07, 95% CI [0.03, 0.11], Bayes *R*^2^ = 0.02, indicating that for each standard deviation increase in PTSD symptom severity, there was a ~7 ms or 6% increase in the magnitude of RB-AB vigilance scores. At a cluster-level of PTSD symptoms, higher levels of alterations in arousal and reactivity (cluster E) were associated with increased RB-AB vigilance after controlling for all other clusters *b* = 0.08, 95% CI [0.02, 0.13]. Surprisingly, higher levels of alterations in arousal and reactivity were also uniquely associated with RB-AB avoidance *b* = 0.07, 95% CI [0.02, 0.12].

With regards to trait anxiety, there was evidence of an association between trait anxiety and avoidance at baseline, *b* = 0.05, 95% CI [0.01, 0.09], Bayes *R*^2^ = 0.01 with a corresponding ~6 ms or 5% increase in RT for each standard deviation increase in trait anxiety. However, the evidence did not support an association between trait anxiety and vigilance *b* = 0.02, 95% CI [−0.01, 0.05], Bayes *R*^2^ = 0.005.

#### 3.2.3. Change in RB-AB by Time and Treatment Condition

There were meaningful main effects of time with reductions in RB-AB vigilance at mid-treatment, *b* = −0.18, 95% CI [−0.22, −0.14], reflecting a 15% decrease in vigilance or ~15 ms change, and posttreatment, *b* = −0.11, 95% CI [−0.15, −0.07], reflecting a 10% decrease in vigilance or ~9 ms change. Similarly, there was a main effect of time with meaningful reductions in RB-AB avoidance at mid-treatment *b* = −0.18, 95% CI [−0.23, −0.13], reflecting an 18% decrease in avoidance or ~19ms change, and posttreatment *b* = −0.07, 95% CI [−0.12, −0.01], reflecting a 9% decrease in avoidance or ~10 ms change. There was no main effect of the nonpersonalized ABM condition on vigilance, *b* = −0.04, 95% CI [−0.10, 0.02], or avoidance, *b* = −0.05, 95% CI [−0.13, 0.02]. Similarly, there was no main effect of the Personalized ABM condition on vigilance, *b* = −0.05, 95% CI [−0.11, 0.01], or avoidance, *b* = −0.06, 95% CI [−0.13, 0.01].

**Figure 3:**
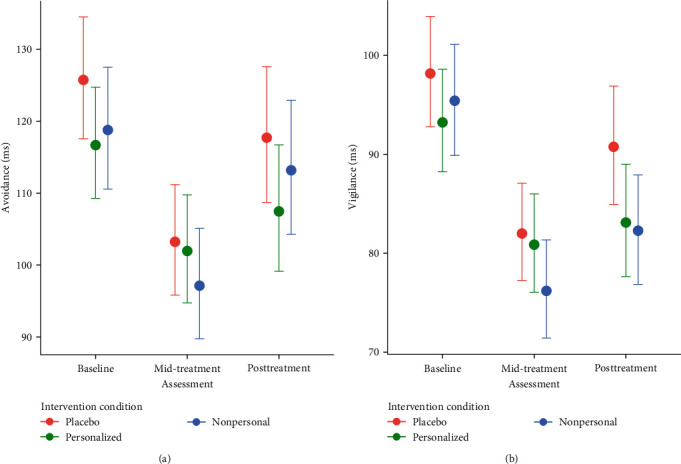
Results from models testing the interaction between assessment and intervention conditions on avoidance/vigilance attention bias. Results highlight large decreases in attention bias for both vigilance and avoidance at mid-treatment, with some increases at posttreatment: (a) time × condition on avoidance bias; (b) time × condition on vigilance bias.

Figure 3 depicts the interaction between time and condition on changes in RB-AB. For vigilance, there was no interaction between time and condition for the personalized intervention condition at mid-treatment, *b* = 0.04, 95% CI [−0.06, 0.13] or posttreatment, *b* = −0.04, 95% CI [−0.14, 0.07]. For avoidance, there was also no interaction between time and condition at mid-treatment, *b* = 0.06, 95% CI [−0.06, 0.18], or posttreatment *b* = −0.02, 95% CI [−0.15, 0.11]. There was also no interaction between the nonpersonalized condition and time at mid-treatment, *b* = −0.04, 95% CI [−0.14, 0.05], or posttreatment, *b* = −0.07, 95% CI [−0.17, 0.03], for vigilance or avoidance at mid-treatment, *b* = −0.00, 95% CI [−0.13, 0.12], or posttreatment, *b* = 0.02, 95% CI [−0.11, 0.15]. Together, these results indicate that RB measures significantly decreased across time (a main effect of time), but the magnitude of change did not significantly differ between treatment conditions (no time x condition interaction).

## 4. Discussion

In our large-scale study among people with clinically significant PTSD symptoms, we found that RB computation yielded vigilance and avoidance scores with both acceptable reliability and meaningful associations with PTSD severity and trait anxiety. Exploratory analyses of PTSD clusters indicated associations of hypervigilance with levels of alterations in arousal and reactivity, which suggests congruence between self-reported symptoms and attention bias. Of note, attentional avoidance was also associated with higher levels of alterations in arousal and reactivity, and not with avoidance. It may be that alterations in arousal and reactivity directly drive attentional avoidance, whereas actively avoiding reminders of the trauma reflects a broader range of cognitive and behavioral mechanisms. If replicated, these findings may have important implications for treatment. In contrast, scores derived using the traditional approach were unreliable and did not exhibit associations with any clinical measures at baseline. Moreover, following attention training with perceived threatening and/or neutral stimuli, we found reductions in RB-AB metrics over time, which was not observed for traditional metrics. For the most part, these findings support the adoption of the RB-AB approach in order to better understand the role of threat-related attention bias in PTSD and to guide the development of more precise interventions to reduce attention bias.

Dominant models of PTSD implicate threat-related attention biases in the maintenance of clinical symptomology [34, 35]. However, recent studies highlighting inconsistent or null associations with PTSD symptoms have produced significant concern about the validity of existing measures of attention biases [16, 19]. An assumption underlying traditional attention bias scores is that a single attention bias score accurately assesses attentional dysfunction in a given individual that is a unidirectional construct (e.g., hypervigilance or avoidance only). However, traditional attention bias measures exhibit very low reliability, which makes it impossible to interpret the mixed results observed across a very large literature. Although it is universally acknowledged that threat-related hypervigilance *and* avoidance are observed in PTSD, these attentional processes are often thought to occur on different timescales or in different individuals [36, 37]. Consistent with previous findings in smaller studies and a recent preprint of a very large-scale study [38], the current study in a large clinical sample (*n* = 640) demonstrated that traditional attention bias measures exhibited poor reliability and failed to detect associations with PTSD symptoms and trait anxiety. In contrast, our results clearly demonstrate that the RB-AB approach improves both the reliability and validity of the threat-related attentional bias in PTSD, even when the dot-probe task is completed entirely remotely on a mobile app. Our findings offer support for a model of PTSD in which vigilance and avoidance represent distinct elements that may jointly serve to maintain symptom severity in the same individuals.

Separating vigilance and avoidance offers a clear way to disentangle two different and sometimes opposing attentional processes thought to play a role in PTSD symptoms. One common model of PTSD is that it involves an exaggerated response to threats, which would suggest that hypervigilance represents the core attention bias in the disorder [34, 39]. However, avoidance of perceived threat is also frequently observed in PTSD and is known to maintain symptoms [36]. The specific role of vigilance to threat in the attention bias literature has been based primarily on initial AB results using the dot-probe and emotional Stroop task with confirmation from a meta-analysis [10]. Our results suggest that disambiguating distinct attentional processes (avoidance and vigilance) may be necessary for properly evaluating and treating attention bias in PTSD. Consideration of approaches that can address both biases simultaneously may be of significant benefit, particularly when considering how to design attention bias-focused interventions for PTSD. One key clinical implication of these more complex results is that if both avoidance and bias are often present among individuals with elevated PTSD symptoms, attention control training that spans both avoidance and vigilance may operate best as an intervention strategy, with some evidence supporting this claim [14, 15]. However, it is also possible that some individuals may be more strongly oriented toward or away from threats, making them better candidates for ABM that addresses a more specific attentional profile for each individual. Further research is needed to determine whether there is underlying heterogeneity in attentional profiles of PTSD and whether interventions can be made more effective if personalized attentional profiles are targeted.

Several limitations are important to note. The number of trials included in the dot probe was relatively low (70) compared with previous studies using the RB-AB approach (160). As a result, it is possible that the smaller proportion of trials available for analyses influenced the observed results. Additionally, it is important to reiterate that all participants completed the dot-probe task on their smartphones. While this may not influence the reliability of the attentional parameters per se, the mode of task administration may nevertheless still influence attention or perhaps attention training in ways that are presently unclear. It is possible that RB-AB indices of attention bias would have even higher reliability scores and better validity if the dot-probe task was administered in a controlled setting and included more trials. The sample was predominantly White, which may limit the generalizability of these results. The salience of the stimuli for the nonpersonalized group was not evaluated and could have played a role in the results. Future research will be necessary to address these issues.

Taken together, these findings suggest that the adoption of RB-AB in the context of ABM research is an important step forward in enhancing our understanding of threat-related attentional bias in PTSD. Indeed, there exists a large body of data that could be reanalyzed with RB-AB to further our understanding of PTSD and other conditions. Such work is needed to both validate our findings and test procedures that can demonstrate the utility of delineating heterogeneous changes in attentional processes through specific attention modification strategies. More widespread use of the RB-AB approach should greatly advance the field and may provide a path forward for making meaningful progress in the implementation of ABM for PTSD.

## Figures and Tables

**Table 1:** Demographic and baseline symptom summary (*N* = 659).

Variable	Mean (SD)
Age (years)	31.90 (9.76)
PTSD checklist	65.94 (14.37)
State-trait anxiety inventory	48.45 (10.17)

	**% (*N*)**

Female	79.5 (524)
Race	
Hispanic/Latino/Mexican	13.1 (86)
Asian	8.2 (54)
African American/Black	12.4 (82)
Caucasian/European American	57.8 (381)
American Indian/Alaskan	0.5 (3)
Hawaiian or other Pacific Islander	0.1 (1)
Race not specified	0.5 (3)
More than 1 race	7.3 (48)
Education	
High school	12.9 (85)
Some college	36.6 (241)
4-year college	31.1 (205)
Postgraduate	19.3 (127)
Not reported	0.2 (1)

**Table 2:** Means (SDs) for reaction time indices across conditions and assessment.

Metric	Placebo			Nonpersonalized			Personalized		
	BL	MID	POST	BL	MID	POST	BL	MID	POST
AVOID	128.75 (69.64)	106.32 (58.43)	126.31 (80.44)	120.83 (68.68)	106.87 (64.82)	117.21 (79.64)	119.76 (57.52)	98.59 (49.84)	119.98 (75.4)
VIG	101.67 (47.47)	83.95 (38.94)	95.33 (45.66)	95.74 (41.74)	84.24 (39.79)	86.82 (40.96)	98.74 (46.16)	78.26 (35.88)	85.94 (38.16)
TRAD	−1.3 (43.73)	−3.05 (42.9)	−3.93 (45.54)	3.37 (43.11)	−1.26 (37.48)	−5.01 (37.8)	−6.44 (45.91)	−1.93 (29.57)	3.27 (46.28)

Abbreviations: AVOID, avoidance response-based attention bias; BL, baseline; MID, mid-treatment; POST, posttreatment; TRAD, traditional attention bias; VIG, vigilance response-based attention bias.

## Data Availability

The R syntax used for Bayesian analyses is available at this link: https://osf.io/kv23r/?view_only = e9b13c9871cb4131b106c8c7f7bfccba. Data are available on request from the senior corresponding author.

## References

[1] Elwood L. S., Hahn K. S., Olatunji B. O., Williams N. L. (2009). Cognitive Vulnerabilities to the Development of PTSD: A Review of Four Vulnerabilities and the Proposal of an Integrative Vulnerability Model. *Clinical Psychology Review*.

[2] Korte K. J., Allan N. P., Gros D. F., Acierno R. (2016). Differential Treatment Response Trajectories in Individuals With Subclinical and Clinical PTSD. *Journal of Anxiety Disorders*.

[3] Naim R., Wald I., Lior A. (2014). Perturbed Threat Monitoring Following a Traumatic Event Predicts Risk for Post-Traumatic Stress Disorder. *Psychological Medicine*.

[4] Bardeen J. R., Orcutt H. K. (2011). Attentional Control as a Moderator of the Relationship Between Posttraumatic Stress Symptoms and Attentional Threat Bias. *Journal of Anxiety Disorders*.

[5] Sipos M. L., Bar-Haim Y., Abend R., Adler A. B., Bliese P. D. (2014). Postdeployment Threat-Related Attention Bias Interacts With Combat Exposure to Account for PTSD and Anxiety Symptoms in Soldiers. *Depression and Anxiety*.

[6] Schoorl M., Putman P., Van Der Werff S., Van Der Does A. J. W. (2014). Attentional Bias and Attentional Control in Posttraumatic Stress Disorder. *Journal of Anxiety Disorders*.

[7] Mazidi M., Vig K., Ranjbar S., Ebrahimi M.-R., Khatibi A. (2019). Attentional Bias and Its Temporal Dynamics Among War Veterans Suffering From Chronic Pain: Investigating the Contribution of Post-Traumatic Stress Symptoms. *Journal of Anxiety Disorders*.

[8] Kaiser D., Jacob G. A., van Zutphen L. (2020). Patients with Borderline Personality Disorder and Comorbid PTSD Show Biased Attention for Threat in the Facial Dot-Probe Task. *Journal of Behavior Therapy and Experimental Psychiatry*.

[9] Wald I., Shechner T., Bitton S. (2011). Attention Bias Away from Threat During Life Threatening Danger Predicts PTSD Symptoms at One-Year Follow-Up. *Depression and Anxiety*.

[10] Bar-Haim Y., Lamy D., Pergamin L., Bakermans-Kranenburg M. J., van IJzendoorn M. H. (2007). Threat-Related Attentional Bias in Anxious and Nonanxious Individuals: A Meta-Analytic Study. *Psychological Bulletin*.

[11] Mogoaşe C., David D., Koster E. H. W. (2014). Clinical Efficacy of Attentional Bias Modification Procedures: An Updated Meta-Analysis. *Journal of Clinical Psychology*.

[12] Kuckertz J. M., Amir N., Boffa J. W. (2014). The Effectiveness of an Attention Bias Modification Program as an Adjunctive Treatment for Post-Traumatic Stress Disorder. *Behaviour Research and Therapy*.

[13] Alon Y., Azriel O., Pine D. S., Bar-Haim Y. (2023). A Randomized Controlled Trial of Supervised Remotely-Delivered Attention Bias Modification for Posttraumatic Stress Disorder. *Psychological Medicine*.

[14] Badura-Brack A. S., Naim R., Ryan T. J. (2015). Effect of Attention Training on Attention Bias Variability and PTSD Symptoms: Randomized Controlled Trials in Israeli and U.S. Combat Veterans. *American Journal of Psychiatry*.

[15] Lazarov A., Suarez-Jimenez B., Abend R. (2019). Bias-Contingent Attention Bias Modification and Attention Control Training in Treatment of PTSD: A Randomized Control Trial. *Psychological Medicine*.

[16] Kruijt A.-W., Parsons S., Fox E. (2019). A Meta-Analysis of Bias at Baseline in RCTs of Attention Bias Modification: No Evidence for Dot-Probe Bias Towards Threat in Clinical Anxiety and PTSD. *Journal of Abnormal Psychology*.

[17] Price R. B., Kuckertz J. M., Siegle G. J. (2015). Empirical Recommendations for Improving the Stability of the Dot-Probe Task in Clinical Research. *Psychological Assessment*.

[18] Schmukle S. C. (2005). Unreliability of the Dot Probe Task. *European Journal of Personality*.

[19] Chapman A., Devue C., Grimshaw G. M. (2019). Fleeting Reliability in the Dot-Probe Task. *Psychological Research*.

[20] Kappenman E. S., Farrens J. L., Luck S. J., Proudfit G. H. (2014). Behavioral and ERP Measures of Attentional Bias to Threat in the Dot-Probe Task: Poor Reliability and Lack of Correlation With Anxiety. *Frontiers in Psychology*.

[21] Iacoviello B. M., Wu G., Abend R. (2014). Attention Bias Variability and Symptoms of Posttraumatic Stress Disorder. *Journal of Traumatic Stress*.

[22] Zvielli A., Bernstein A., Koster E. H. W. (2015). Temporal Dynamics of Attentional Bias. *Clinical Psychological Science*.

[23] Alon Y., Naim R., Pine D. S., Bliese P. D., Bar-Haim Y. (2019). Validity of Attention Bias Variability Indices for Posttraumatic Stress Disorder Research: Evidence From Patient Data. *Journal of Traumatic Stress*.

[24] Todd J., Wilson E., Coutts-Bain D., Clarke P. J. F. (2022). Attentional Bias Variability and Its Association With Psychological and Health Symptoms: A Meta-Analysis. *Neuroscience & Biobehavioral Reviews*.

[25] Kruijt A.-W., Field A. P., Fox E., Lu Z.-L. (2016). Capturing Dynamics of Biased Attention: Are New Attention Variability Measures the Way Forward?. *PLOS ONE*.

[26] Evans T. C., Britton J. C. (2018). Improving the Psychometric Properties of Dot-Probe Attention Measures Using Response-Based Computation. *Journal of Behavior Therapy and Experimental Psychiatry*.

[27] Evans T. C., Bar-Haim Y., Fox N. A., Pine D. S., Britton J. C. (2020). Neural Mechanisms Underlying Heterogeneous Expression of Threat-Related Attention in Social Anxiety. *Behaviour Research and Therapy*.

[28] Niles A. N., Woolley J. D., Tripp P. (2020). Randomized Controlled Trial Testing Mobile-Based Attention-Bias Modification for Posttraumatic Stress Using Personalized Word Stimuli. *Clinical Psychological Science*.

[29] Blevins C. A., Weathers F. W., Davis M. T., Witte T. K., Domino J. L. (2015). The Posttraumatic Stress Disorder Checklist for DSM-5 (PCL-5): Development and Initial Psychometric Evaluation. *Journal of Traumatic Stress*.

[30] Spielberger C. D. (1985). Assessment of State and Trait Anxiety: Conceptual and Methodological Issues. *Southern Psychologist*.

[31] Cristea I. A., Kok R. N., Cuijpers P. (2015). Efficacy of Cognitive Bias Modification Interventions in Anxiety and Depression: Meta-Analysis. *The British Journal of Psychiatry*.

[32] Niles A. N., O’Donovan A. (2018). Personalizing Affective Stimuli Using a Recommender Algorithm: An Example With Threatening Words for Trauma Exposed Populations. *Cognitive Therapy and Research*.

[33] Bürkner P.-C. (2017). brms: An R Package for Bayesian Multilevel Models Using Stan. *Journal of Statistical Software*.

[34] Ehlers A., Clark D. M. (2000). A Cognitive Model of Posttraumatic Stress Disorder. *Behaviour Research and Therapy*.

[35] Vasterling J. J., Brailey K., Constans J. I., Sutker P. B. (1998). Attention and Memory Dysfunction in Posttraumatic Stress Disorder. *Neuropsychology*.

[36] Adenauer H., Pinösch S., Catani C. (2010). Early Processing of Threat Cues in Posttraumatic Stress Disorder—Evidence for a Cortical Vigilance-Avoidance Reaction. *Biological Psychiatry*.

[37] Kimble M. O., Hyatt A. S. (2018). Vigilance/Avoidance to Expected and Presented Stimuli in Trauma Survivors: An Eye-Tracking Study. *Journal of Trauma & Dissociation*.

[38] Xu I., Passell E., Strong R. (2022). No Evidence for Consistent Reliability Across 36 Variations of the Emotional Dot Probe Task in 9,000 Participant.

[39] Bryant R. A., Felmingham K. L., Kemp A. H. (2005). Neural Networks of Information Processing in Posttraumatic Stress Disorder: A Functional Magnetic Resonance Imaging Study. *Biological Psychiatry*.

